# Implementation of Virtual Interactive Cases for Pharmacy Education: A Single-Center Experience

**DOI:** 10.1177/87551225231224627

**Published:** 2024-01-31

**Authors:** Karen Cameron, Erin Cicinelli, Cindy Natsheh, Miranda So, Gordon Tait, Henry Halapy

**Affiliations:** 1 Leslie Dan Faculty of Pharmacy, University of Toronto, Toronto, ON, Canada; 2 University Health Network, Toronto, ON, Canada; 3 St. Michael’s Hospital, Unity Health, Toronto, ON, Canada

**Keywords:** clinical pharmacy, education, simulation, clinical practice, continuing education, pharmaceutical care

## Abstract

Patient case simulation software are described in pharmacy education literature as useful tools to improve skills in patient assessment (including medication history-taking and physical assessment), clinical reasoning and communication, and are typically well-received by students and instructors. The virtual interactive case (VIC) system is a web-based software developed to deliver deliberate practice opportunities in simulated patient encounters across a spectrum of clinical topics. This article describes the implementation and utilization of VIC in the undergraduate curriculum at one Canadian pharmacy school. Methods: At our facility, the use of VIC was integrated across the training spectrum in the curriculum, including core and elective didactic courses and practice labs, experiential learning, interprofessional education, and continuing education. Its use was evaluated through student and instructor surveys and qualitative student interviews). VIC is easy to navigate and created a positive and realistic learning environment. Students identified that it enhanced their ability to identify relevant patient information, accurately simulated hospital pharmacy practice and thereby helped them to prepare for their upcoming experiential courses. The use of VIC has expanded beyond its original intended purpose for individual student practice to become a valuable addition to pharmacy undergraduate education. Future plans include ongoing development of cases and exploration of further uses of VIC within the didactic curriculum, for remediation in experiential courses, and for pharmacist continuing education.

## Introduction

Numerous patient case simulation software have been described in pharmacy education literature as useful tools to improve skills in patient assessment, clinical reasoning, and communication.^1-9^ These tools can improve student performance in medication history-taking and physical assessment skills, and they have been well-received for their value-add and utility in pharmacy education.^1,2,4-6,10-12^ Most importantly, simulation software provides the opportunity for deliberate practice, which is considered a crucial step in the development of expertise in one’s field.^13^ The virtual interactive case (VIC) system is a web-based software developed to deliver deliberate practice opportunities in simulated patient encounters across a spectrum of clinical topics (http://pie.med.utoronto.ca/vic/VIC_content/VIC_pharmacyCases.html). The virtual interactive case is a proprietary software product that was developed in 2011 locally at our university and utilized in our healthcare curricula (eg, medicine in 2011, nursing in 2014). The software program was designed to create cases about patient clinical encounters, including functional assessments, physical exams, ordering/utilizing appropriate laboratory tests, consulting with specialists, reviewing diagnostic tests to determine a problem or diagnosis, and its treatment. The program provides debriefing, including steps taken that were essential, actions that were inappropriate, and actions done in an incorrect order.^14^ It is this use in other curricula, and VIC’s potential advantages, that interested us in adapting for teaching in our university’s pharmacy curriculum.

The VIC system provides an environment to practice foundational pharmacy skills, such as information-gathering, patient assessment, problem-solving, and critical thinking while providing specific and timely feedback tailored to the student’s performance. VIC offers several unique advantages over traditional paper cases, including better representation of the healthcare environment.^15^ A limiting factor of paper cases (which are often used in our undergraduate pharmacy curriculum) is that relevant information needed to identify patient issues is provided directly to students. In VIC, users must extract information that is perceived to be pertinent to the case and discern irrelevant details (rather than being presented with all the required information). Automated feedback is provided and tailored to the user’s selected responses during the information-gathering stage, identifying the drug therapy problem (DTP), and care plan implementation steps. VIC may be adapted for any pharmacy practice setting (community, ambulatory, and inpatient) and can seamlessly integrate multiple disease states simultaneously into one patient case allowing for a diverse breadth of patient complexities, thereby providing authentic patient encounters. In addition, the entire VIC process is timed to simulate real-life time constraints. VIC contains time and cost scores to help students gauge the efficiency of their assessment skills and discourage accessing non-essential or irrelevant items. Feedback about accessing non-essential or irrelevant items is provided at the end of each case summary. The ease of use and positive experience by users suggests that VIC has potential to be used as a tool to deliver continuing education to enhance pharmacist’s clinical practice.^10,16^ This article describes the implementation and evolution of VIC in the undergraduate curriculum at one Canadian pharmacy school, including lessons learned for pharmacy curricula.

## Application of VIC in Pharmacy Education

**Figure 1.**
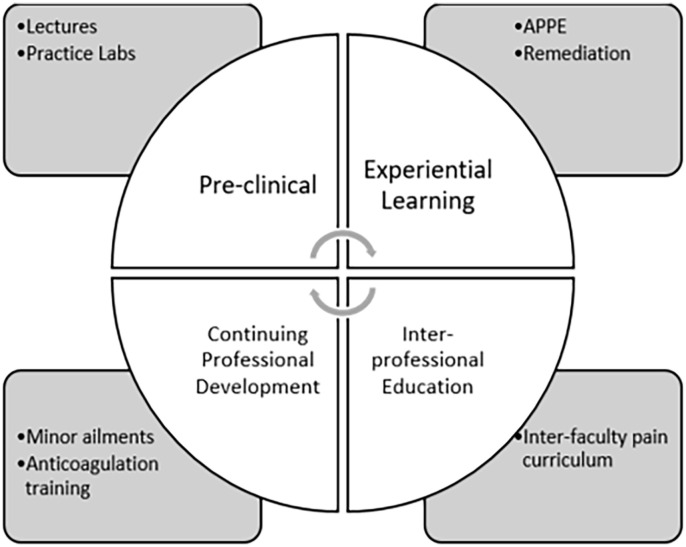
Application of VIC across different stages of pharmacy education. Abbreviations: APPE: Advanced Pharmacy Practice Experience; VIC, virtual interactive case.

At our faculty, VIC has been implemented across the training spectrum in the curriculum: (1) pre-clinical learning, (2) experiential learning, (3) interprofessional education, and (4) continuing education (Figure 1). For pre-clinical learning, VIC cases are incorporated into several core and elective courses (eg, endocrinology, nephrology, and antimicrobial stewardship) where students use them to self-assess their content knowledge and ability to interpret findings of investigations, such as lab results and diagnostic imaging reports. Examples of VIC clinical scenarios include treatment of *Clostridium difficile* infection, insulin management, and medication management in patients with chronic kidney disease. In pharmacy skills labs, students review the VIC patient charts to gather pertinent information, identify the DTP(s), and formulate care plans. They then proceed to communicate their recommendations directly to a standardized clinician. VIC has been used to help facilitate interprofessional case discussions where VIC stems have been used as the basis of the discussion. In undergraduate clinical rotations, students use VIC to review or practice their patient care process prior to working-up real patients, which is particularly useful for preparation for specialty rotations, such as antimicrobial stewardship. In addition, VIC is used for remediation for students struggling in Advanced Pharmacy Practice Experience (APPE) rotations. In this setting, faculty members have students work on VIC cases concurrent to their APPE or use them as part of academic support to improve students’ chance of success prior to completion of a supplemental APPE rotation. The cases are selected from the VIC pharmacy page and chosen to address the areas in need of practice for the individual student. A summary of the courses in which VIC is used in our curriculum is found in Table 1. Finally, for continuing education, VIC may be used to provide illustrative case examples that supplement lecture, practice examples, and training materials (eg, for minor ailment prescribing and anticoagulation management training). In all of these settings, the cases can be used in multiple ways. In the practice labs and some pharmacotherapy courses, they are primarily used for information-gathering, where the student goes on to identify the DTPs and create their own care plans that are then presented to the clinical instructor either verbally or in writing. In these cases, the instructors would grade the students in line with the specific course rubrics. In other courses, VIC is used to its full capability, where the students pick the correct DTP and care plan items from the options provided. Students are then given feedback in the case by the software on the answers chosen, why they were correct or incorrect as well as feedback on their process, including time and cost, and which components of the case they missed or reviewed unnecessarily. When used for APPE remediation, we have also created companion activities for the cases, which may include questions from health care team members, “what if” scenarios, critical appraisal activities and interprofessional team activities. Table 2 provides some examples of these activities.

**Table 1.** Courses Where VIC Is Used in the Curriculum.

Course code	Course name	Description
PHM202H	Pharmacotherapy 3 (Endocrine, Nephrology, Urology)	Cases used for pharmacotherapy workups and care planning (in lieu of paper cases)
PHM206H	Medication Therapy Management-3 (MTM-3)	Cases used to mimic a hospital patient chart for information-gathering
PHM251H	Early Practice Experience-2 (EPE-2)	Cases not used explicitly in this course, but VIC used in PHM206 to better prepare for this experience
PHM305H	Medication Therapy Management-4 (MTM-4)	Cases used to mimic a hospital patient chart for information-gathering
PHM383H	Antimicrobial Stewardship	Cases used for in class discussions
PHM385H	Diabetes Care	Cases used for pharmacotherapy workups and care planning (in lieu of paper cases)
PHM401/402/424H (and others)	Advanced Pharmacy Practice Experiences	Used for extra practice for patient care workups while on APPE rotations or for remedial work

Abbreviations: APPE, Advanced Pharmacy Practice Experience; VIC, virtual interactive case.

**Table 2.** Examples of Companion Activities Used in Remediation.

Activity	Sample
Drug information questions	The patient’s wife requests more information on smoking cessation products for her husband. How do you respond verbally? The RN asks if 2 medications can be combined in the same IV bag. Write a written response.
“What if” scenarios	What if the patient was 29 years old? What if the patient was pregnant? How does your plan change?
Critical appraisal	Present a journal article review of one of the references used in your assessment.
Interprofessional team	Identify members of the health care team to whom your patient may benefit from a referral. The team dietician asks for information on which of the patient’s medications can be crushed. Write a response.
Continuity of care	Create a best possible medication discharge plan for this patient if he were to be discharged today.

## Evaluation of VIC

VIC has been evaluated in a number of ways. The original hospital pharmacy case template and cases were assessed for usability and student feedback in a qualitative study. Five main themes were derived from the student interviews in this study: VIC facilitated students’ skills in information-gathering; students learned from the built-in, real-time, formative feedback; students had a fun and positive learning experience; VIC cases were realistic; and the VIC system was user-friendly.^15^ This early feedback set the stage for further case development and use of VIC for the educational purposes described previously.

When VIC was first introduced into a second-year skills lab course (Medication Therapy Management-3 (MTM3), we sought student and clinical instructor feedback as the format and flow of the lab was very different than a typical lab. A summary of the student feedback is found in Tables 3 and 4. Most students in the first cohort (62%) indicated that they would have liked more than the 20 minutes allotted to review the VIC prior to presenting their findings and recommendations to the clinical instructor. More students (75%) in a subsequent cohort, who were given the same amount of time to review the case plus an additional 5 minutes to organize their thoughts prior to the interaction with clinical instructors found the allotted 20 minutes to be adequate. As one of the goals of including VIC in the lab was to allow students an opportunity to gather information in a manner that forced them to select necessary information to identify the DTP and create a plan, we found this to be important feedback. Despite the time pressure felt by students, performance was similar to other non-VIC labs in both cohorts. Students were asked whether they felt that use of VIC in this course would assist them with their future hospital experiential courses, and 49% of students in the first cohort and 76% in the second cohort responded favorably (Table 4). A clinical instructor feedback survey indicated that they felt students generally performed well on the lab, and interaction time between the instructor and the student was sufficient (Tables 5 and 6).

**Table 3.** Student Feedback on Time Allotted to Review the VIC in the Lab (20 Minutes) (MTM-3).

Cohort	Number of students responded/number of students in class	Too little, N (%)	Just right, N (%)	Too much, N (%)
2018	55/236	34 (62)	30 (36)	1 (2)
2019	227/241^a^	57 (25)	161 (71)	0 (0)

Abbreviation: MTM, Medication Therapy Management.

a In 2019, students completed feedback survey prior to leaving the lab rather than an optional online feedback survey in 2018.

**Table 4.** Student Feedback on VIC Preparing Them for Hospital Practice (MTM-3).

Cohort	Number of students responded/number of students in class	Strongly agree, N (%)	Agree, N (%)	Neutral, N (%)	Disagree, N (%)	Strongly disagree, N (%)
2018	55/236	2 (4)	25 (45)	16 (29)	10 (18)	2 (4)
2019	227/241	23 (10)	150 (66)	49 (22)	5 (2)	0 (0)

Abbreviation: MTM, Medication Therapy Management.

**Table 5.** Clinical Instructor Feedback “Students Generally Performed Well on the Lab” (MTM-3).

Cohort	Number of CIs	Strongly agree, N (%)	Agree, N (%)	Neutral, N (%)	Disagree, N (%)	Strongly disagree, N (%)
2018	22/22	1 (4.5)	15 (68.2)	3 (1.4)	2 (9.1)	1 (4.5)
2019	14/19	3 (21.4)	10 (71.4)	0 (0)	1 (7.1)	0 (0)

Abbreviation: MTM, Medication Therapy Management.

**Table 6.** Clinical Instructor Feedback on Time Allotted for Student Interaction (MTM-3).

Cohort	Number of CIs	Too little (%)	Just right (%)	Too much (%)
2018	22/22	0 (0)	22 (100)	0 (0)
2019	14/19	0 (85.7)	12 (100)	2 (14.3)

Abbreviation: MTM, Medication Therapy Management.

To assess further the impact of VIC cases on the preparation of students for hospital practice, 136 students who completed their Early Practice Experience-2 (EPE-2) placements in a hospital pharmacy setting were invited to participate in an interview to explore the contribution of VIC to their perceived readiness for a hospital placement. Thirteen students who agreed to participate were interviewed by one of the VIC project team members. These semi-structured interviews were conducted in person or over the telephone using an interview guide (Appendix A). These students had also been exposed to VIC cases in a second-year pharmacotherapy course as well as the practice lab course discussed above. Although the 13 students represented approximately 10% (13/136) of all eligible students who were invited to participate, it was determined that data saturation occurred at the eighth participant, as no new themes were identified in subsequent interviews during data analysis. Themes derived from these interviews revealed some similarities to the original qualitative study^15^ as they also found VIC software easy to navigate. Additional themes included: VIC enhances students’ ability to identify relevant patient information; VIC accurately simulated hospital pharmacy practice, thereby helping to prepare students for their rotations; and students recommended an expanded role of VIC in the curriculum.

When VIC was subsequently introduced into the third-year skills lab (Medication Therapy Management–4, MTM-4) to assess students’ skills at discharge medication reconciliation similar feedback was obtained. Sixty-four percent felt that they needed more time to review the case, 90% felt that it would help prepare them for future APPEs, and almost all students (98%) successfully passed the lab (Tables 7 and 8).

**Table 7.** Student Feedback on Time Allotted to Review the VIC in the Lab (20 Minutes) (MTM-4).

Cohort	Number of students responded/number of students in class	Too little, N (%)	Just right, N (%)	Too much, N (%)
2019	203/225	130 (64)	73 (36)	0 (0)

Abbreviation: MTM, Medication Therapy Management.

**Table 8.** Student Feedback on VIC Preparing Them for Hospital Practice (MTM-4).

Cohort	Number of students responded/number of students in class	Strongly agree, N (%)	Agree, N (%)	Neutral, N (%)	Disagree, N (%)	Strongly disagree, N (%)
2019	203/225	14 (7)	116 (57)	65 (32)	8 (4)	0 (0)

Abbreviation: MTM, Medication Therapy Management.

A formal evaluation on the use of VIC in the interfaculty pain curriculum has not been undertaken due to the volume of metrics already assessed in this multi-faculty undertaking. Anecdotal feedback from interprofessional facilitators in the first year the cases were used included suggestions for making the program accessible on tablets and mobile devices. This is now possible with the updated version of the software.

Feedback collected from experiential education Course Coordinators indicates that VIC assists students with information-gathering and patient care process skills as well as for creating thorough care plans. Using VIC within APPEs allows for students to build confidence outside of the rotation site by practicing individually with multiple cases. In addition, coordinators indicate that a case can also be used to form the foundation for other skills, including answering hypothetical drug information questions that could arise from team members, presenting a verbal case summary, suggesting referrals that may be indicated for the patient and creating best possible medication discharge plans.

Similar feedback on the use of VIC has been garnered from multiple users and student cohorts suggesting that not only is VIC practical and versatile to use, but it also helps prepare learners for practice.

## Implications and Significance for Practice

The VIC software was developed to help students bridge the skill gap during the transition from the classroom to practice setting, with the intention to enhance students’ preparedness for their experiential rotations and clinical practice.^14^ Our experience suggests that VIC is a useful tool to support improved patient data-gathering skills, better preparation for practice by mimicking the practice environment more closely than traditional paper cases, and improved critical thinking. The VIC model of case writing is also versatile, in that it is adaptable to many patient types and complexities, clinical scenarios, and practice environments.

The use of VIC in various Doctor of Pharmacy courses was well-received by students, and the overall positive response from using virtual patient cases is consistent with previous literature and other simulation programs.^1,2,5,7,10,17,18^ Our experiences highlight the realism of the VIC software in simulating electronic medical records and practice environments.

The use of multiple VICs in a particular topic area with different patient characteristics is achievable. This unique feature enables implementation into pharmacotherapy or skills-based courses and allows for easy modification and updating of cases from year to year. In addition, cases of any level of complexity (eg, one simple DTP to several concurrent DTPs) and any therapeutic area can be created. Furthermore, VIC can be written for any practice environment (eg, inpatient hospital, ambulatory care, primary care, community pharmacy), expanding its use for preparing students to common practice environments encountered by pharmacy students. Our findings suggest that the use of VIC can also be expanded to other pharmacy faculties as well as continuing education programs for pharmacists. These realistic practice cases could help with not only knowledge acquisition, but continued skills development. As a starting point, a selection of pharmacy VICs is freely available on the VIC website.

## Limitations and Lessons Learned

The process of developing VIC for pharmacy students required a significant collaborative effort from a wide array of faculty members (experiential, practice-based, and course-based). This collaborative process could be expanded to include members to enhance the case inventory suitable for practicing pharmacists. Although VICs are relatively easy to create using the case manual, it does require technical support to export the cases and host them on a web page for access. This expertise would typically be found within educational institutions. An initial financial outlay for a VIC software license ($5000) may also be a limitation for some sites. As with other simulation programs, VIC is a bridge to real-life practice, but VICs are not a substitute for experiential learning with real patients. However, the use of VIC is less costly than live simulations with standardized patients and is easier to deploy. A summary of the advantages and disadvantages of VIC based on our experience is found in Table 9.

**Table 9.** Summary of VIC Advantages and Disadvantages.

Advantages	Disadvantages
Easy to use for participants	Initial learning required of user
Easy to write new cases	Initial training or review of case writing guide required
Less expensive than standardized actors or other patient simulation software	Expensive relative to paper cases. One-time license cost $5000
Cases can be hosted on a webpage for easy access	Requires technical support to host cases on a web page
Cases can be adapted to a variety of patient complexities or multiple disease state	Cases are not as authentic as live patient cases
Cases are easily modified to develop a bank of cases on one specific disease state to enable multiple opportunities to practice (deliberate practice)	Cases may need to be updated to reflect new evidence/practice change
Cases include images of laboratory values, medication lists or other reports as required	
Cases can be adapted for any pharmacy practice setting	
Can be adapted for many levels of pharmacy learners (eg, undergraduate student, remediation, continuing pharmacist education)	
Helps develop patient assessment skills as students need to discern between relevant and irrelevant information	
Provides feedback on accessing non-essential/irrelevant data	
Automated feedback tailored to user’s selected response	Some students may require more feedback than automated system gives
Provides time and cost of intervention scores → helps students gauge efficiency of their assessment skills	

## Conclusion

The use of VIC has expanded beyond its original intended purpose for individual student practice to become a valuable addition to pharmacy undergraduate education. Future plans include ongoing development of cases and exploration of further uses of VIC within the didactic curriculum, for remediation in experiential courses, and for pharmacist continuing education.

## Appendix A

### Interview Guide—Use of VIC Cases in Second-Year Curriculum

Thank you for agreeing to be interviewed for this project. During your second year of pharmacy school, you used VICs in 2 courses: PHM202, also known as PCT-3: Endocrinology, Nephrology and Urology, and PHM206, also known as MTM-3. I will be asking you questions to gain an understanding of your perspectives on the use of VICs in these courses how/if you feel they prepared you for your institutional EPE-2 rotation this summer.

You may choose not to answer a question, stop the interview, or withdraw from the interview at any time. The interview will be recorded and then transcribed. In the transcription, you will be identified by an ID number to keep your identity anonymous. I also ask that you not mention your name, the location of your rotation and your preceptor name during the interview to maintain anonymity.

This interview should take approximately 20 to 30 minutes to complete.

Do you have any questions before we begin?

#### Demographic information

1.  Did you complete your EPE-2 rotation at either a hospital or a family health team?
If student answers yes, proceed with remaining interview questions.
If student answers no: verify location of rotation and confirm that it does NOT meet criteria for institution and then end the interview.
2. In which rotation block did you complete your EPE-2? (blocks 1, 2, or 3?)
For interviewer, reference in case you need to jog the student’s memory: block 1 = May 6 to June 7; block 2 = June 10 to July 12; block 3 = July 15 to August 16

#### Questions about VIC

1. Did you have any experience with VICs before the use of VIC in the fall term of 2018 (PHM202H—PCT-3: Endocrinology, Nephrology, and Urology)?
Expectation is that students will say no. If they say yes: Describe your prior experience with VIC.
2. VIC was introduced in the fall term of 2018 in PHM202 (PCT3). Do you recall using VICs in that course?
If prompt required: Paper cases were used for workshops 1 to 4 (BPH, ED, Urinary incontinence, contraception, menopause) and then for workshops 5 to 7 (insulin dosing, nephrology cases, and integrated case).
2a. What was your impression of the use of VIC at the time? Did you note any differences between the cases used for the VIC workshops and the cases used for the other workshops?
Potential prompts if required: time to work through case, amount of information provided, how realistic the cases were?
3.  VIC was also used in the winter term of 2019 in PHM206 (MTM-3). Do you recall using the VIC in that course?
If prompt required: VIC was used in lab 6 for the UTI topic
3a. What was your impression on the use of VIC at the time? Did you note any differences between the VIC lab and the other labs?
Potential prompts if required: type of information-gathering? How realistic was the case? Type of interaction?
4. In general do you find VICs to be:
User-friendly? Why or why not?
Fun? Why or why not?
5. Thinking about your experiences on the use of VIC in these 2 courses, do you think they helped prepare you for your institutional EPE-2 experience?
If yes or any positive hint:
a. In what way did they help prepare you? or What about VICs helped prepare you?
b.  In which course, PHM202 or MTM-3, do you think VIC was more helpful in preparing you? Why?
c. Is there a time during your rotation where you were engaged in an activity and you remembered the use of VIC in one of the courses and you thought: “I remember doing something like this before.” If so, what were you doing at the time?
If no: Why do you think that was the case?
6. Do you have any suggestions as to how we could use VIC to help prepare students for EPE-2? Prompt if necessary:
a. Are there other ways in PHM202 or MTM-3 that VIC could be used?
b. Are there other settings or courses in which VIC could be used?
c. Are there specific clinical topics that you think VIC could be used to teach?
7. Is there anything else you would like to say about the use of VICs in your second year courses? About VIC in general?
